# Editorial: Pulmonary hypertension associated with congenital heart disease

**DOI:** 10.3389/fped.2023.1217010

**Published:** 2023-05-24

**Authors:** Siyi He, Hong Qian, Xingbo Xu

**Affiliations:** ^1^ Department of Cardiovascular Surgery, General Hospital of Western Theater Command, Chengdu, China; ^2^ Department of Cardiovascular Surgery, West China Hospital, Sichuan University, Chengdu, China; ^3^ Department of Cardiology and Pulmonary, University Medical Center Göttingen, Göttingen, Germany

**Keywords:** pulmonary hypertension, congenital heart disease, pulmonary arterial smooth muscle cells, inspiratory muscle training, healthcare-associated infection, omics

**Editorial on the Research Topic** Pulmonary hypertension associated with congenital heart disease

Pulmonary hypertension (PH) is a serious hemodynamic disorder, threatening the quality and expectancy of life. According to the newly published European Society of Cardiology (ESC) and European Respiratory Society (ERS) guideline, PH is classified into five clinical subtypes, defined by an increased mean pulmonary arterial pressure over 20 mmHg (1). Pulmonary hypertension associated with congenital heart disease (PH-CHD) belongs to the first main category and usually caused by congenital malformation with systemic-pulmonary shunt. At present, standardized treatment for PH-CHD remains controversial with a multitude of regulatory mechanisms needed to be explored. This Research Topic focuses on both clinical and basic research about PH-CHD, providing novel perspectives and viewpoints on tackling existing challenges.

Proliferation of pulmonary arterial smooth muscle cells (PASMCs) is considered to be an essential basis for PH formation, driven by a variety of cytokines, such as platelet-derived growth factor (PDGF). In the study of Qin et al. the role of ataxia telangiectasia mutated (ATM) in PDGF-BB-induced proliferation of PASMCs was explored. The optimal level of PDGF-BB could accelerate the formation of PH by activating the limited amount of reactive oxygen species (ROS) and inhibiting the phosphorylation of ATM. Although high concentration of PDGF-BB did not exert the proliferative effect on PASMCs, the ATM inhibitor showed additional positive effects under the excessive levels of ROS. This finding confirmed restoration of ATM function in preventing the pathological proliferation of PASMCs, which may be a novel target for treatment of PH.

Muneuchi et al. comprehensively summarized the importance of pulmonary vascular resistance (Rp) and capacitance (Cp) on evaluating pulmonary circulation in children with PH-CHD. Pulmonary arterial input impedance is formed when heart ejects blood against pulmonary arterial load, of which Rp and Cp are essential determinants. The relationship between Rp and Cp presents universal hyperbolic. Cp rather than Rp can reflect an early stage of reduced effective pulmonary vascular beds following impaired recruitment and distensibility of the pulmonary capillaries. It is emphasized that the combined assessment of Rp and Cp is necessary in patients with PH-CHD who have different disease conditions. Measures targeting the increase in Cp will be helpful for determining the effectiveness of pulmonary vasodilators.

Supervised exercise training is recommended as Class I in PH patients with optimal drug treatment by the 2022 ESC/ERS guideline, of which inspiratory muscle training (IMT) is a simple and practicable physical therapy approach. Luo et al. performed a systematic review and meta-analysis to explore the safety and feasibility of isolated IMT in PH patients. Four studies comprising 80 patients were included. Results showed that IMT could improve maximal inspiratory pressure, maximal expiratory pressure and 6 min walk distance. No significant difference was found in pulmonary function and quality of life. The most prominent issue in the promotion of IMT is the lack of standardized protocol. Different training methods can lead to different therapeutic effects. Some patients are even not suitable for IMT, because excessive training will aggravate the burden of heart and lung. That's the very reason why some clinical studies have opposite clinical outcomes.

Persistent pulmonary hypertension of the newborn (PPHN) is another common type of PH in children, characterized with high morbidity and mortality rate. Lin et al. established a nomogram prediction score system by using the plasma pH value, septicemia, and abnormal pregnancy history to facilitate individualized prediction of early death in newborns with PPHN. Although this model has a satisfactory accuracy with the area under the ROC curve (AUC) of 0.737, single-centric data and lack of external validation are major limitations. There is still a long way for it to be applied in clinical practice.

Li et al. analyzed the clinical characteristics of healthcare-associated infection (HAI) in patients with PH-CHD in a tertiary hospital of western China. Data from 1,129 patients revealed a per-case HAI rate of 3.37% and a per-thousand day HAI rate of 25.62. In infected patients, those with CHD-PAH were younger and prone to receive surgical corrections. Surgical correction for cardiac malformations is an essential therapeutic method of PH-CHD. Children are at increased risk of infection after surgery for the reasons like cardiopulmonary bypass related inflammation response, undeveloped function of multi-organs, immature immune system, and so on. The authors mentioned that one of limitations in the manuscript was that they neglected the change of management strategies since the pandemic of COVD-19. Actually, some valuable prevention lessons from COVID-19 can be used for the control of HAI in patients with PH-CHD. A lower risk of infection will inevitably result in a better clinical prognosis.

Zhao et al. presented a detailed description of the latest advances on omics and PH-CHD, including genomics, transcriptomics, epigenomics, proteomics, metabolomics, and multi-omics integration. With the rapid development of high throughput technology, omics research has become a hot topic in biological and medical field in recent years. Through omics, we can get a complete picture of PH-CHD from the genetic to epigenetic level, which is helpful to quickly find pathogenic molecules and their corresponding signaling pathways. On the other hand, the changes of omics in blood contribute to seek potential novel biomarkers to predict the occurrence and progression of disease. Application of omics technology provides a new strategy for accurate diagnosis and treatment of PH-CHD.

Preventing the progression of pulmonary vascular remodeling and keeping patients at low risk are therapeutic goals for PH-CHD. Although the clinical efficacy has been greatly improved with the modification of surgery operations and the presence of targeted drugs, there are still a portion of patients needing a lifelong treatment. Comprehensive and reasonable application of various treatment means including medication, surgery, rehabilitation, family education and so on will be the future research direction to achieve the individualized management of PH-CHD patients. Focusing on a precision health, artificial intelligence algorithm with the aid of multi-omics technology will facilitate diagnosis and risk stratification of PH-CHD on the basis of big data and multicenter data collection.
